# [Expression of Concern] Histone deacetylase inhibitors induce in human hepatoma HepG2 cells acetylation of p53 and histones in correlation with apoptotic effects

**DOI:** 10.3892/ijo.2026.5896

**Published:** 2026-05-20

**Authors:** D. Carlisi, B. Vassallo, M. Lauricella, S. Emanuele, A. D'anneo, E. Di Leonardo, P. Di Fazio, R. Vento, G. Tesoriere

Int J Oncol 32: 177-184, 2008; DOI: 10.3892/ijo.32.1.177

Following the publication of the above paper, it was drawn to the Editor's attention by a concerned reader that, regarding the western blot analyses shown in Fig. 2A and B on p. 179, the control β-actin blots in Fig. 2A were very similar to a section of the control blots in Fig. 2B, albeit with some horizontal and vertical resizing of the bands in question, even though the affected lanes of the gel highlighted different experimental treatments.

The authors were contacted by the Editorial Office to offer an explanation for this apparent anomaly in the presentation of the data in this paper, and we are awaiting their response. Owing to the fact that the Editorial Office has been made aware of potential issues surrounding the scientific integrity of this paper, we are issuing an Expression of Concern to notify readers of this potential problem while the Editorial Office continues to investigate this matter further.

